# Tincture of Iodine in the Treatment of Porrigo

**Published:** 1846-05

**Authors:** 


### TINCTURE OF IODINE IN THE TREATMENT OF PORRIGO.

Dr. F. W. Todd, of Port Gibson, Miss., informs us that he has found the *Tincture of Iodine*, employed locally, "invaluable in the treatment of that obstinate disease, Porrigo." He has used it in all forms of the disease whether mild or severe, and in no instance has it failed to effect a cure. In the more active forms, poultices and fomentations preceded the application of the iodine.

